# The Use of 3D-Printed Space Maintainers: Clinical Outcomes and Parental Satisfaction

**DOI:** 10.7759/cureus.115136

**Published:** 2026-08-25

**Authors:** Faisal E. A. A. A. Alkandari

**Affiliations:** 1 Dentistry, Kuwait Ministry of Health, Kuwait City, KWT

**Keywords:** deciduous, dental care, evidence gaps, patient satisfaction, tooth, workflow

## Abstract

Space maintainers (SMs) have traditionally been the treatment of choice for preventing space loss following the premature loss of primary teeth. The advent of digital dentistry has revolutionized dental care with the introduction of 3D-printed space maintainers. A systematic literature review was conducted to synthesize the existing evidence on the use of 3D-printed SMs, their clinical outcomes, and parental satisfaction. The literature review was conducted in accordance with PRISMA (Preferred Reporting Items for Systematic Reviews and Meta-Analyses) guidelines. A predefined search strategy led to the selection of 12 studies published between 2019 and 2025. The data were analyzed narratively. An enhanced patient experience during impression-taking through a digital workflow was a significant factor contributing to patient satisfaction. The survival and failure rates of 3D-printed SMs were dependent on the material properties used in the intervention. Resin-based SMs were more prone to failure due to decementation, whereas metal-based SMs were more mechanically durable. Regarding clinical outcomes, gingival health consistently improved; however, this improvement did not persist beyond six months. No other clinical outcomes, such as Plaque Index (PI), dimensional stability, or soft tissue trauma, were reported. Evidence regarding parental satisfaction was scarce, although overall satisfaction was enhanced, mainly due to the digital impression workflow. This research gap may be attributed to the nascent stage of these interventions; however, long-term validation and efficacy studies are warranted to fully realize the potential of these technologies.

## Introduction and background

In pediatric dentistry, one of the most prevalent and challenging phenomena is premature loss of primary teeth driven by dental caries and trauma [1]. This impacts the developing dental arch by inducing loss of space, midline deviation, and asymmetric molar relationship, among others [2,3]. The impact becomes more severe in the case where deciduous teeth are lost, leading to malocclusion of the permanent dentition. Studies report that the premature loss of primary teeth increases the occurrence of orthodontic treatment, while also observing that losing a primary molar reduces space for the next permanent teeth [4,5].

Loss of space has been a significant drawback of premature tooth loss, which can be rectified by using space maintainers (SMs). These help in keeping the arch shape intact and are available in different kinds, such as removable or fixed SMs [6]. Conventional band and loop (CB&L) SMs have traditionally been employed, which are effective and cost-saving. However, several limitations of CBLSMs have been reported, including multiple visits, appliance dislodgement, metal allergy, gingival inflammation, and plaque accumulation [7]. Moreover, they are technically sensitive appliances and may trigger the gag reflex, particularly in young or uncooperative children [8]. Solder breakage and cement loss are additional disadvantages of these appliances [6].

The advent of technology has transformed traditional practices into digital dentistry, which involves the utilization of intraoral scanning (IOS) [9], computer-aided design (CAD) [10], and additive manufacturing (3D printing) [11]. The digital workflow enabled by these technologies has helped overcome the limitations associated with conventional impression-making by facilitating the use of digital impressions, which are precise, time-efficient, and more comfortable for children. These technologies also reduce chairside time [12]. The validation of these technologies is currently a major focus of the literature, with a growing body of evidence demonstrating their efficiency within a relatively short period.

A fracture strength analysis conducted by Metkar et al. (2024) reported greater mechanical stability for 3D-printed band and loop space maintainers (3D-BLSMs), with a fracture strength of 308.53 N, compared with 130.85 N for conventional band and loop space maintainers (CBLSMs). This underscores the superiority of these modern technologies [13]. When the survival rates of 3D-BLSMs were compared with those of conventional SMs, a nine-month randomized controlled trial (RCT) reported a 77.4% survival rate for 3D-BLSMs. These findings suggest that 3D-printed SMs may offer greater durability and comfort over the long term [14]. Another RCT studying survival rates and patient/parent satisfaction reported 65% survival rates of 3D-BLSMs, while the satisfaction levels were comparable between 3D-BLSMs and CBLSMs [15].

The field of 3D-printed SMs is still in its nascent stage, and several aspects require more robust evidence from larger RCTs and cohort studies. The advantages of this technology in terms of technical robustness and survival rates have been widely reported across studies [16,17]; however, patient-centered outcomes remain inadequately addressed [18]. Despite digital workflows being increasingly integrated into clinical practice, there is a lack of research evaluating their use in streamlining the diagnostic and management processes in pediatric dentistry [19]. Studies assessing “satisfaction,” whether among patients or parents, are either scarce or based on non-standardized measures [14,15]. Furthermore, no study has specifically evaluated parental satisfaction with 3D-printed SMs [16,20].

In comparison to other dental specialties where parental satisfaction is measured as a critical parameter of clinical success [21], parental satisfaction in the field of 3D-printed SMs has emerged as a critical research gap. Parental satisfaction is crucial because parents’ perceptions of these technological advancements and digital workflows may influence the course of treatment, as parents are the primary caregivers for their children and are responsible for making health-related decisions [22]. Given this research gap, a comprehensive systematic review is warranted to evaluate the current state of research on 3D-printed SMs, particularly their clinical outcomes and parental satisfaction. This literature review seeks to synthesize current scholarship on 3D-printed SMs, including their use, clinical outcomes, and parental satisfaction. The research questions addressed in this literature review are as follows: 1. What clinical outcomes regarding the use of 3D-printed SMs in children and adolescents are reported across the literature? 2. How satisfied are the parents with the results of 3D-printed SMs?

## Review

Methodology

Study Design

A systematic review methodology was employed to assess the current evidence base regarding 3D-printed SMs, their use, clinical outcomes, and parental satisfaction. The literature review was conducted in accordance with the PRISMA (Preferred Reporting Items for Systematic Reviews and Meta-Analyses) guidelines, ensuring a transparent, reproducible, and robust review process.

Eligibility Criteria

A predefined eligibility criterion was developed using the PCC (Population, Concept, Context) framework to evaluate the articles (Table 1). The publication period for eligible studies ranged from 2019 to 2025. Only studies published in English were included. Since the field of 3D-printed SMs is still emerging and lacks a robust evidence base from large cohorts and RCTs, case studies were also included to provide a comprehensive understanding of the efficacy of this intervention, while acknowledging the potential heterogeneity of the available data.

**Table 1:** PCC framework for the selection of eligible studies PCC: Population, Concept, Context; SM: space maintainer; CAD: computer-aided design

Component	Inclusion criteria	Exclusion criteria
Population	Children and adolescents (≤ 18 years) who have undergone placement of a fixed SM following the premature loss of a primary tooth	Studies where the population includes adults, or SMs are placed for reasons other than premature primary tooth loss (e.g., trauma, congenital agenesis)
Concept	The use of 3D-printed SMs fabricated using a digital workflow (including intraoral scanning, CAD, and additive manufacturing). The review will focus on two core concepts: 1. Clinical outcomes: Survival rate, gingival health, Plaque Index, caries incidence on abutment teeth, dimensional stability/space loss, and incidence of soft tissue trauma. 2. Parental satisfaction: Levels and drivers of satisfaction, including the experience of the digital workflow (e.g., comfort, convenience, number of appointments)	Removable SMs. SMs fabricated using conventional (non-digital) methods or other non-3D-printed digital methods (e.g., milled). Studies that do not report on at least one pre-specified clinical or satisfaction-related outcome
Context	Clinical studies conducted in any setting (e.g., university hospital, private practice) that report on the outcomes of 3D-printed SMs. Studies can be of any design that involves clinical follow-up	In-vitro (laboratory-based) studies, technical procedure descriptions without patient outcomes, and studies published only as conference abstracts without sufficient methodological or results detail. Studies not published in English
Study Type	Original primary research, including Randomized Controlled Trials (RCTs), cohort studies (prospective and retrospective), case-control studies, and cross-sectional studies with follow-up data. Case series will also be included to capture a wider evidence base for this emerging technology.	Narrative reviews, systematic reviews, meta-analyses, editorials, and commentaries

Search Strategy

The literature was searched using academic databases including PubMed, Scopus, Google Scholar, Web of Science, and Cochrane Central Registry of Controlled Trials (CENTRAL). The search strategy encompassed keyword combinations derived from database-specific vocabulary terms (e.g., MeSH terms for PubMed). These include “Space Maintainers,” “Tooth, Deciduous,” “Tooth Loss,” “Child,” “Pediatric Dentistry,” “3D Printing,” “Printing, Three-Dimensional,” “Computer-Aided Design,” “Digital Dentistry,” “Digital Impression,” “Intraoral Scanning,” “Additive Manufacturing,” “Patient Satisfaction,” “Parental Satisfaction,” “Treatment Outcome,” “Survival Rate,” “Gingival Health,” “Plaque Index”. Search strings employed in different databases are presented in Table 2. The language filter was applied to include only English studies.

**Table 2:** Search string for literature retrieval

Database	Controlled vocabulary terms	Search string (adapted for each database's syntax)
PubMed	"Anemia, Sickle Cell"[Mesh] "Psychology"[Mesh] "Mental Health"[Mesh] "Quality of Life"[Mesh] "Social Stigma"[Mesh] "Child"[Mesh] "Adolescent"[Mesh] "Young Adult"[Mesh]	("Space Maintenance" OR "Space Maintainer" OR "Space Maintainers" OR "Tooth Loss"[Mesh] OR "Tooth, Deciduous"[Mesh]) AND ("3D Printing" OR "Three-Dimensional Printing" OR "Additive Manufacturing" OR "Computer-Aided Design"[Mesh] OR "Digital Dentistry"[Mesh] OR "Intraoral Scanner" OR "Intraoral Scanners") AND ("Patient Satisfaction"[Mesh] OR "Parental Satisfaction" OR "Treatment Outcome"[Mesh] OR "Survival Rate" OR "Gingival Health" OR "Plaque Index") AND ("Child"[Mesh] OR "Pediatric Dentistry"[Mesh] OR "Adolescent"[Mesh])
Scopus	None	("Space Maintenance" OR "Space Maintainer" OR "Space Maintainers" ) OR ( "Tooth Loss" OR "Deciduous Tooth" ) AND ( "3D Printing" OR "Three-Dimensional Printing" OR "Additive Manufacturing" OR "Computer-Aided Design" OR "Digital Dentistry" OR "Intraoral Scanner" OR "Intraoral Scanners" ) AND ( "Patient Satisfaction" OR "Parental Satisfaction" OR "Treatment Outcome" OR "Survival Rate" OR "Gingival Health" OR "Plaque Index" ) AND ( child OR children OR adolescent OR adolescents OR pediatric ) OR ( "Child" OR "Adolescent" OR "Pediatric Dentistry" )
Web of Science	None	TS=("Space Maintenance" OR "Space Maintainer" OR "Space Maintainers" OR "Tooth Loss" OR "Deciduous Tooth") AND TS=("3D Printing" OR "Three-Dimensional Printing" OR "Additive Manufacturing" OR "Computer-Aided Design" OR "Digital Dentistry" OR "Intraoral Scanner" OR "Intraoral Scanners") AND TS=("Patient Satisfaction" OR "Parental Satisfaction" OR "Treatment Outcome" OR "Survival Rate" OR "Gingival Health" OR "Plaque Index") AND TS=(child OR children OR adolescent OR adolescents OR pediatric OR "Young Adult")
Cochrane CENTRAL	None	("Space Maintenance" OR "Space Maintainer" OR "Space Maintainers" OR "Tooth Loss" OR "Deciduous Tooth") AND ("3D Printing" OR "Three-Dimensional Printing" OR "Additive Manufacturing" OR "Computer-Aided Design" OR "Digital Dentistry" OR "Intraoral Scanner" OR "Intraoral Scanners") AND ("Patient Satisfaction" OR "Parental Satisfaction" OR "Treatment Outcome" OR "Survival Rate" OR "Gingival Health" OR "Plaque Index") AND (child OR children OR adolescent OR adolescents OR pediatric)
Google Scholar	Not Applicable	"3D printed space maintainer," "parental satisfaction," "clinical outcomes," pediatric dentistry

Study Selection

**Figure 1:**
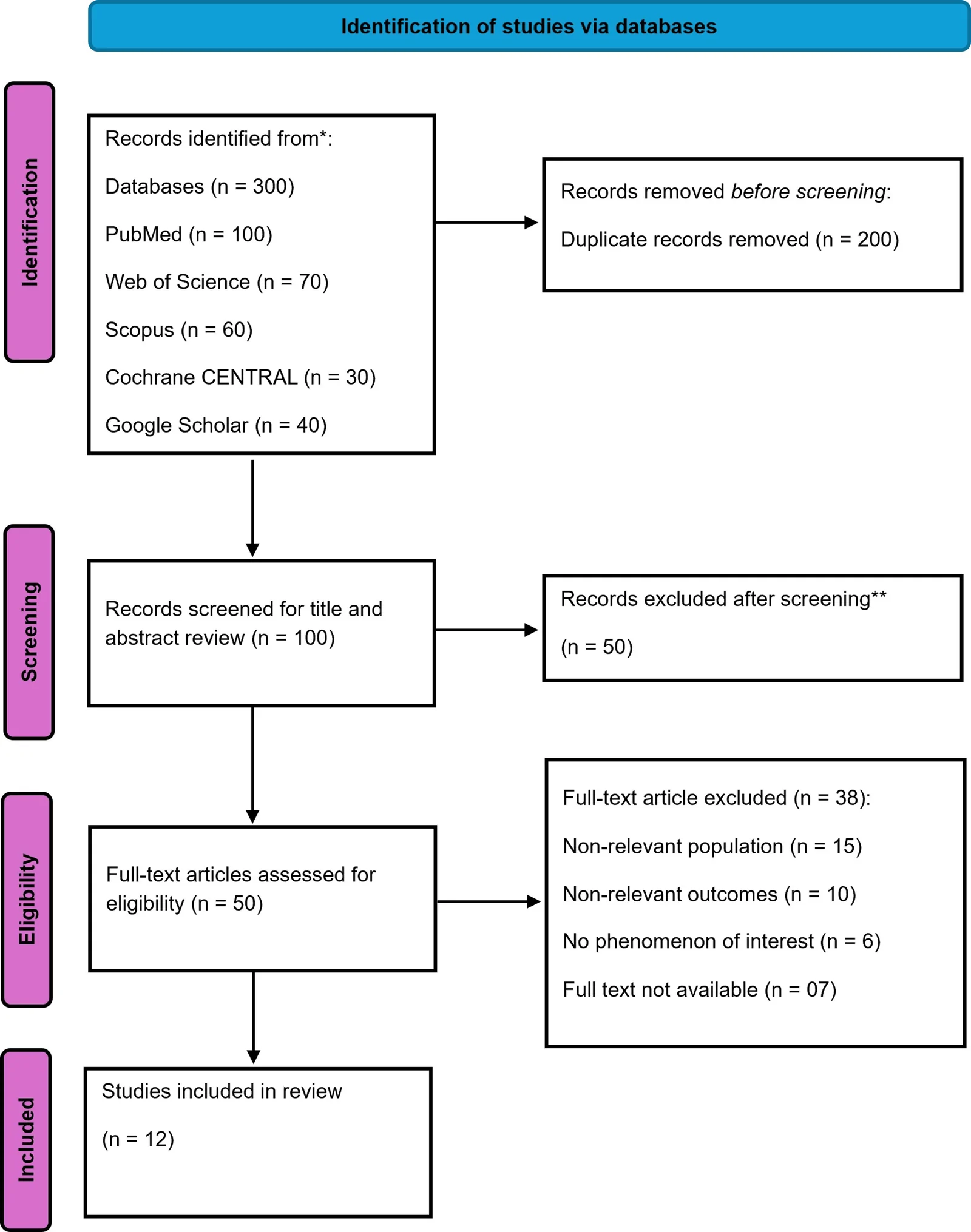
PRISMA flow diagram depicting the study selection PRISMA: Preferred Reporting Items for Systematic Reviews and Meta-Analyses

The total number of articles extracted from electronic databases was 300, of which 200 duplicates were identified and removed. During the initial screening phase, titles and abstracts were reviewed, resulting in the exclusion of 50 irrelevant articles. The remaining 50 articles were then screened according to the predefined eligibility criteria based on the PCC framework (Table 1), and the studies were assessed for eligibility and full-text availability. Thirty-eight studies were excluded for not meeting the eligibility criteria. The remaining 12 articles met the predefined criteria and were included in the final synthesis. A visual representation of the screening process is provided in the PRISMA flowchart (Figure 1).

Data Extraction and Analysis

A data extraction form was developed and used to systematically extract relevant information from the included studies, including study characteristics, population and intervention details, outcome data, key findings, and methodological information. The detailed data extraction criteria are presented in Table 3. In case of disagreements, a third reviewer was consulted, and consensus was reached through mutual discussion. The collected data were analyzed using a narrative synthesis approach to interpret the findings and provide an in-depth synthesis of the available literature on the use of 3D-printed space maintainers, their clinical outcomes, and parental satisfaction.

**Table 3:** Summary of data extraction variables RCT: randomized controlled trial; SLA: stereolithography; CAD: computer-aided design

Category	Variables for extraction
Study identification and characteristics	Author(s), publication year, journal, country of study, funding source, type of study (e.g., RCT, cohort, case series)
Study methodology	Primary objective/aim, study design, data collection period, follow-up duration, conflict of interest statement
Population and context	Sample size, age range and mean age, teeth replaced, criteria for space maintainer placement, clinical setting (e.g., university hospital, private practice)
Intervention details	Type of 3D-printed space maintainer (e.g., band and loop), 3D printing technology (e.g., SLA), resin material used, software for CAD, type of intraoral scanner
Outcome data: clinical	Survival rate/time to failure, reasons for failure, gingival health index and scores, plaque index and scores, caries incidence on abutment teeth, dimensional stability/space loss (in mm), incidence of soft tissue trauma, other complications
Outcome data: parental Satisfaction	Method of assessment (e.g., validated questionnaire, custom survey, interview), timing of assessment, quantitative satisfaction scores, qualitative themes and/or direct quotes, specific drivers of satisfaction/dissatisfaction (e.g., comfort, aesthetics, convenience, digital impression experience, number of appointments, perceived durability)
Authors' conclusions and relevance	The authors' main conclusions on efficacy and satisfaction, identified strengths and limitations of the 3D-printed technology, identified future research needs, and relevance to clinical practice

Quality Assessment

All included studies were critically appraised for methodological rigor and risk of bias to ensure the synthesis was based on high-quality evidence. The studies were grouped according to their study design, and an appropriate quality assessment tool was selected for each study type. The appraisal tools used were as follows: the Cochrane Risk of Bias 2 (RoB 2) Tool for randomized controlled trials (RCTs), the Risk of Bias in Non-Randomized Studies of Interventions (ROBINS-I) for non-randomized comparative studies, and the Joanna Briggs Institute (JBI) Critical Appraisal Checklist for case reports and case series. The studies were classified as having “High,” “Moderate,” or “Low” quality based on their individual assessments. Overall, the selected evidence base demonstrated substantial methodological rigor, strengthening the validity of the synthesis. Consolidated quality appraisal ratings are presented in Table 4.

**Table 4:** Quality assessment of the included studies RCT: randomized controlled trial; RoB 2: Revised Cochrane risk-of-bias tool for randomized trials; ROBINS-I: Risk Of Bias In Non-randomized Studies - of Interventions; JBI: Joanna Briggs Institute

Study	Type of study	Appraisal tool used	Overall quality
Kapoor et al., 2025 [15]	RCT	RoB 2	High
Thakur et al., 2024 [14]	RCT	RoB 2	High
Nedeljkovic et al., 2025 [23]	RCT	RoB 2	High
Khatab et al., 2025 [24]	RCT	RoB 2	Moderate
Cengiz and Karayilmaz, 2024 [25]	Non-randomized comparative study	ROBINS-I	Low
Joshi et al., 2024 [26]	Case report	JBI checklist	Moderate
Lee, 2023 [27]	Case report	JBI checklist	High
Mladenovic et al., 2025 [28]	Case report	JBI checklist	High
Pawar, 2019 [29]	Case report	JBI checklist	Moderate
Rathi et al., 2023 [30]	Case report	JBI checklist	Moderate
Vij and Reddy, 2020 [31]	Case report	JBI checklist	Moderate
Yangdol et al., 2023 [32]	Case report	JBI checklist	Moderate

Results

Study Characteristics

The predefined search strategy identified 12 eligible studies, comprising four RCTs [14,15,23,24], one comparative study with randomized sampling [25], one case series [30], and six case reports [26-32]. Geographically, most studies were conducted in India (7/12), followed by Serbia, Korea, and other countries. As 3D-printed technologies are relatively new, the reported sample sizes were small, ranging from 20 to 70 participants across the included studies. Follow-up durations ranged from three to 22 months, with study outcomes most commonly assessed at six or nine months. Study characteristics are summarized in Table 5.

**Table 5:** Study characteristics RCT: randomized controlled trial; PMMA: polymethyl methacrylate; Co-Cr: cobalt-chromium; DMLS: direct metal laser sintering; CAD/CAM: computer-aided design and computer-aided manufacturing; FDM: fused deposition modeling

Study	Country	Study design	Sample size (3D)	Type of 3D-printed SM	Material/technology	Follow-up	Clinical outcomes reported	Parental satisfaction assessed
Kapoor et al. (2025) [15]	India	RCT	20	Band and loop	Not specified	9 months	Survival, gingival health	Yes (5-point Likert)
Thakur et al. (2024) [14]	India, Indonesia, UAE	RCT	31	Band and loop	Titanium alloy (micro laser sintering)	9 months	Survival, gingival health	Yes (5-point Likert)
Cengiz and Karayilmaz (2024) [25]	Turkey	Comparative clinical	32 (38 appliances)	Band and loop	Titanium alloy (laser sintering)	6 months	Survival, gingival health, Plaque Index	Yes (patient survey on impressions)
Khatab et al. (2025) [24]	Egypt	RCT (split-mouth)	20 appliances	Band and loop	PMMA resin	6 months	Survival, gingival health	No
Nedeljkovic et al. (2025) [23]	Serbia	RCT	15	Functional (KOS&MET)	Co-Cr metal alloy	6 months	Survival, bite force, masticatory performance	No
Rathi et al. (2023) [30]	India	Case series	2	Band and loop	Titanium alloy	6 months	Anecdotal survival	No
Yangdol et al. (2023) [32]	India	Case report	1	Band and loop	Co-Cr alloy (DMLS)	Not stated	Anecdotal survival	Yes (implied)
Lee (2023) [27]	Korea	Case report	1	Band and loop (reverse)	Milled zirconia (CAD/CAM)	22 months	Anecdotal survival	Yes (implied)
Pawar (2019) [29]	India	Case report	1	Band and loop	Co-Cr alloy (laser sintering)	3 months	Anecdotal survival	No
Vij and Reddy (2020) [31]	USA	Case report	1	Lingual holding arch	Cast metal (on 3D-printed model)	Not stated	Anecdotal survival	No
Joshi et al. (2024) [26]	India	Case report	1	Band and loop (on 3D-printed model)	Cast metal (on FDM-printed model)	3 months	Anecdotal survival	No
Mladenovic et al. (2025) [28]	Serbia	Case report	1	Functional (KOS&MET)	Co-Cr metal alloy	12 months	Survival, masticatory performance	No

Synthesis of Clinical Outcomes

Survival rate and failure: Considerable variability in survival rates was reported across the literature on 3D-printed SMs, primarily associated with the manufacturing process and the materials used. The majority of studies used either resin-based polymers or metal-based additive manufacturing techniques. Survival rates varied from 65% for 3D-BLSMs to 85% for conventional SMs [15]. Similarly, failure rates of 66% for laser-sintered (LS) SMs compared with 38% for conventional SMs were reported at or within six months of follow-up [25]. Poor retention rates were also reported for 3D-printed resin appliances at six months, with retention decreasing to 80% compared with 95% for conventional SMs.

Contrary to these findings, higher survival rates have been reported with metal-based additive manufacturing. Thakur et al. (2024) reported greater long-term durability of 3D-printed SMs, with a survival rate of 77.4% after nine months compared with 51.6% for conventional SMs [14]. Different types of 3D-printed SMs, such as metal functional maintainers, have also demonstrated survival rates of 87%, as reported by Nedeljkovic et al. Similarly, a case study conducted by Mladenovic et al. reported no instances of detachment or breakage after 12 months of follow-up in patients treated with 3D-printed SMs [28]. Decementation was identified as one of the major factors influencing the survival and failure rates of 3D-printed SMs, as corroborated by Cengiz and Karayilmaz, with no cases of solder breakage reported [25].

Gingival health and Plaque Index: Studies consistently reported positive effects of 3D-printed SMs on gingival health. Khatab et al. (2025) reported a reduction in gingival inflammation, assessed using the Marginal Gingival Index, with statistically significant differences observed in the 3D-printed SM group at one month (median: 1.50 vs. 2.25) and three months (median: 1.25 vs. 2.00) of follow-up. However, these improvements were not sustained at six months [24]. A similar trend was reported by Kapoor et al., although the difference was statistically non-significant, with “mild gingivitis” (0.1-1.0) persisting across the intervention groups. Cengiz and Karayilmaz (2024) further reported that although plaque and gingival indices remained low in both groups, their values at six months showed an increase compared with baseline measurements [25].

Other clinical outcomes: Apart from gingival health, no other specific clinical outcomes, such as caries, dimensional stability, or soft tissue trauma, were systematically assessed across the included studies. The study by Cengiz and Karayilmaz was the only study to evaluate the Plaque Index. Interestingly, masticatory performance was evaluated in studies involving functional SMs [23,28], and an improvement in masticatory performance following SM placement was also reported [28].

Synthesis of Parental and Patient Satisfaction

There is an evident research gap regarding parental satisfaction; in our synthesis of the 12 selected studies, only three attempted to assess satisfaction [14,15,25]. In these three studies, satisfaction was evaluated quantitatively. Thakur et al. reported 90% patient satisfaction, while Kapoor et al. assessed long-term satisfaction over a nine-month period and reported a mean score of 3.55/5 on a 5-point Likert scale. The reported satisfaction levels were comparable to those observed with conventional SMs [15].

The driving factor for high satisfaction across the evidence base was the impression experience of 3D-printed SMs, where 97% of patients labelled it “easy and fun,” and all of them were ready to experience it again (Cengiz Karayilmaz). These findings, in comparison to their conventional counterparts, were 27% and 6%, respectively. Commonly reported elements leading to dissatisfaction with conventional SMs were “nausea”, “discomfort”, and “taste/odor of the foreign body” [15]. Two other elements influencing satisfaction were reduced chairside time and aesthetics. While plaque was attributed as a dissatisfaction entity [15], patients also reported mild pain immediately after 3D-BLSMs; however, it quickly subsided, achieving the ultimate goal of maintaining aesthetics [14].

Summary of Evidence From Case Reports

3D-printed SMs are a relatively new and emerging technology, with most of the available evidence derived from case studies [26-31], which are considered lower-quality evidence for evaluating the efficacy of sophisticated interventions. However, these studies remain valuable for demonstrating the feasibility and potential clinical utility of newer interventions in specific clinical settings. Case studies involving 3D-printed SMs have consistently reported positive outcomes, such as reduced chairside time and improved clinical utility, particularly in challenging pediatric populations, including children with autism [32] and ADHD [30]. One of the commonly reported mechanical advantages of 3D-printed SMs is their one-piece construction, which eliminates the need for solder joints. Additionally, Lee reported enhanced satisfaction and improved aesthetics during a 22-month follow-up of a 3D-printed SM intervention [27].

Discussion

This literature review explored the newly emerging 3D-printed SMs, their clinical outcomes, and parental satisfaction. The current state of evidence from our findings has helped us characterize the advantages and challenges of this intervention in pediatric dentistry, particularly in the digital era. Our findings clearly demonstrate that 3D-printed SMs as a technology have immense potential; however, their efficacy might increasingly be dependent on the material and design of the appliance. Patient-centered outcomes and research in terms of parental satisfaction are scarce, which impedes the adoption of this technology. The interesting aspect of our findings is that, more than the technology in question, the associated digital workflow is a crucial factor positively influencing patient experience of 3D-printed SMs.

Current Clinical Landscape of 3D-Printed Space Maintainers

One of the research questions of this study was to assess the clinical outcomes of 3D-printed SMs. Among the specified health outcomes, the available evidence addressed only survival rates and gingival health. A marked difference in survival rates was observed based on the material used for 3D-printed SMs, namely resin-based or metal-based materials. The major drawback of resin-based SMs was decementation. This highlights the fact that the materials used to fabricate these 3D-printed appliances are critical determinants of their clinical success. Recent in vitro studies support this finding, demonstrating that despite having adequate flexural strength, resin-based SMs may lack long-term durability [31]. To enhance bonding durability, the broader literature also recommends the use of materials with appropriate flexibility along with adequate surface treatment [33].

In comparison with resin-based SMs, metal-based 3D-printed SMs appear to be more favorable, primarily because of their higher survival rates, which may be attributed to their “single-unit” construction that eliminates the need for soldering. Conversely, a major reason for the failure of CBLSMs is the potential failure of solder joints [34]. Modern practices such as additive manufacturing using suitable metals may therefore help overcome some of the limitations of conventional SMs. Fracture strength analyses have demonstrated greater mechanical durability of 3D-printed SMs compared with their conventional counterparts [35].

The majorly improved health or clinical outcome reported is gingival health via consistent use of 3D-printed SMs. This provides 3D-printed SMs with another significant advantage over conventional ones that frequently deal with plaque accumulation [36]. This improvement may be the by-product of using CAD-supported precision, enabling a marginal fit and smoother surface finish [37].

Patient and Parental Satisfaction

Assessing parental satisfaction with the use of 3D-printed SMs was another objective of this study. However, there was a substantial lack of research addressing this aspect. Furthermore, a striking finding from our review is that satisfaction levels were associated more with the digital workflow and application process than with the appliance itself. This finding is supported by the broader literature, with patients preferring intraoral scanning for impression-taking over conventional methods [25], as it is considered more comfortable for pediatric patients [38].

General pediatric dentistry is increasingly benefiting from the integration of digital dentistry. During the placement of conventional SMs, young children often report feelings of “nausea” and “discomfort,” which can negatively affect their overall treatment experience. To address these concerns, digital workflows have been introduced and have been well received by children. This approach is also beneficial for children with neurodevelopmental conditions such as autism and ADHD [30,32]. Our review identified plaque control [15] and positive digital experiences as important determinants of long-term satisfaction, with the latter contributing to improved patient comfort.

Addressing the Contradictions and Identifying the Research Gaps

The variability of survival rates might be driven by the use of separate technologies like polymer printing and metal sintering. It is recommended that these technologies be employed in light of material science. This review underscores that it is vital for the audience to include adequate detail about the materials and technology when reporting about technological advancements such as 3D-printed SMs. The central missing aspect highlighted in our study is the inadequate focus on parental satisfaction in the literature. Even in those studies where satisfaction parameters are investigated, non-validated instruments are used. Parental satisfaction modulates children's response to treatment adherence, making it an indispensable part of pediatric research.

Limitations and future recommendations

This review provides valuable evidence regarding the advantages and limitations of 3D-printed SMs in the literature. However, several limitations need to be addressed. Initially, the lack of high-quality evidence (RCTs/larger cohorts) with smaller sample sizes poses generalizability issues, and the smaller number of studies leads to heterogeneity of the data. Secondly, inclusion of case reports, while essential for mapping the evidence for a nascent technology, is equivalent to low-quality evidence. Future research should focus on conducting long-term studies with larger sample sizes, focusing on the patient-centered outcomes of 3D-printed SMs, comparative studies of metal-based SMs against conventional SMs, and the impact of materials, their biocompatibility, and the design of the appliance.

## Conclusions

This literature review synthesized the available evidence on the use of 3D-printed SMs, their clinical outcomes, and associated parental satisfaction. The primary finding of our study is that the patient experience facilitated by the digital workflow is a significant driver of satisfaction. It was also observed that the material used for the intervention plays a more important role in determining clinical efficacy. Metal-sintered SMs were found to be more mechanically durable and associated with higher survival rates than resin-based 3D-printed SMs. While these technologies may help overcome traditional limitations such as gingival inflammation and solder breakage, they also present their own unique challenges.
